# Rapid Evolution of Coral Proteins Responsible for Interaction with the Environment

**DOI:** 10.1371/journal.pone.0020392

**Published:** 2011-05-25

**Authors:** Christian R. Voolstra, Shinichi Sunagawa, Mikhail V. Matz, Till Bayer, Manuel Aranda, Emmanuel Buschiazzo, Michael K. DeSalvo, Erika Lindquist, Alina M. Szmant, Mary Alice Coffroth, Mónica Medina

**Affiliations:** 1 Red Sea Research Center, King Abdullah University of Science and Technology (KAUST), Thuwal, Saudi Arabia; 2 European Molecular Biology Laboratory, Heidelberg, Germany; 3 Section of Integrative Biology, School of Biological Sciences, University of Texas at Austin, Austin, Texas, United States of America; 4 School of Natural Sciences, University of California Merced, Merced, California, United States of America; 5 Department of Anesthesia, UCSF School of Medicine, University of California San Francisco, San Francisco, California, United States of America; 6 Department of Energy Joint Genome Institute, Walnut Creek, California, United States of America; 7 Center for Marine Science, University of North Carolina Wilmington, Wilmington, North Carolina, United States of America; 8 Graduate Program in Evolution, Ecology and Behavior and Department of Geology, State University of New York at Buffalo, Buffalo, New York, United States of America; King Abdullah University of Science and Technology, Saudi Arabia

## Abstract

**Background:**

Corals worldwide are in decline due to climate change effects (e.g., rising seawater temperatures), pollution, and exploitation. The ability of corals to cope with these stressors in the long run depends on the evolvability of the underlying genetic networks and proteins, which remain largely unknown. A genome-wide scan for positively selected genes between related coral species can help to narrow down the search space considerably.

**Methodology/Principal Findings:**

We screened a set of 2,604 putative orthologs from EST-based sequence datasets of the coral species *Acropora millepora* and *Acropora palmata* to determine the fraction and identity of proteins that may experience adaptive evolution. 7% of the orthologs show elevated rates of evolution. Taxonomically-restricted (i.e. lineage-specific) genes show a positive selection signature more frequently than genes that are found across many animal phyla. The class of proteins that displayed elevated evolutionary rates was significantly enriched for proteins involved in immunity and defense, reproduction, and sensory perception. We also found elevated rates of evolution in several other functional groups such as management of membrane vesicles, transmembrane transport of ions and organic molecules, cell adhesion, and oxidative stress response. Proteins in these processes might be related to the endosymbiotic relationship corals maintain with dinoflagellates in the genus *Symbiodinium.*

**Conclusion/Relevance:**

This study provides a birds-eye view of the processes potentially underlying coral adaptation, which will serve as a foundation for future work to elucidate the rates, patterns, and mechanisms of corals' evolutionary response to global climate change.

## Introduction

Reef-building corals (Cnidaria: Hexacorallia: Scleractinia) are of fundamental ecological significance in tropical and sub-tropical shallow marine environments as they form the most important components of coral reefs. These organisms are sensitive to the current rising global seawater temperatures [1] resulting in increased frequencies of mass coral bleaching events, which in turn have caused severe declines in live coral cover [2]. To this end, much effort has been committed to assessing factors affecting overall vulnerability and resilience of reef corals [3], [4], [5], [6]. Additional work has been devoted to the identification of stress-responsive genes [7], [8], [9], [10], [11], [12]. However, few studies have looked into the genetic makeup of corals that might help determining to what extent corals are able to respond to increasing disturbances and stress by means of evolutionary adaptation [13]. Thompson and van Woesik [14] found that corals at sites with a high-frequency of thermal stress displayed less bleaching than at other sites, despite being exposed to a greater level of stress. The authors suggest that bleaching resistance is most likely a consequence of rapid directional selection following an extreme thermal event, i.e. corals are able to respond adaptively from the pool of standing genetic variation. Other studies have shown that multicolored fluorescent proteins display a considerable amount of adaptive, convergent, and parallel evolution in corals [15], [16]. Schwarz *et al.* [17] characterized a ferritin in *Acropora millepora* and *Acropora palmata* that displays signs of positive selection. Hayes *et al.* detected adaptive evolution in tachylectins [18].

Adaptive evolution, at the molecular level, is characterized by an excess of nonsynonymous nucleotide substitutions (*d_N_*) in comparison to synonymous ones (*d_S_*) [19], [20], [21], [22], [23]. If this is the case, the so-called *d_N_/d_S_* ratio becomes >1, and the gene of interest may be under positive selection. Note that a single important amino acid change may be sufficient to demonstrate positive selection. However, methods for site-specific adaptive evolution analyses require multiple pair-wise comparisons, thus inclusion of sequence data from multiple species. Evolutionary screens are designed in a way that orthologs in a designated group of genes are ‘scanned’ for elevated *d_N_/d_S_* ratios. These screens provide a powerful way to identify, in a single effort, many candidate genes that are potentially subject to positive selection. Circumstantially, there is no *a priori* requirement to know the function of the protein, a factor that is particularly beneficial in non-model organisms such as corals. However, lack of annotation cannot be considered a difficulty exclusively associated with non-model species as the number of genes without any significant sequence similarity to genes of other species in any eukaryotic genome surveyed so far seems to be about 10–20% [24], [25], [26]. It is assumed that these genes represent lineage-specific adaptations of the species under study as they not only lack sequence similarity to genes or proteins in other organisms, but also display a narrow phylogenetic distribution. There is no general agreement or rule, but usually, proteins which do not show any sequence similarity in BLASTp searches with cut-off values of E<10^−5^ or E<10^−10^ have been denoted as so-called taxonomically-restricted genes (TRGs) [27], and have been hypothesized to provide one of the sources of phenotypic diversity [28], [29], [30]. TRGs are synonymously referred to as lineage-specific genes [29]. A recent screen by Sunagawa *et al.* [31] identified a family of small, cysteine-rich proteins (SCRiPs) that appear to be restricted to Hexacorallia. A study in *Hydra* identified Periculin-1, a peptide that has strong bactericidal activity and at present no identifiable orthologs in sequence databases [32].

The amount of positive Darwinian selection has not yet been systematically surveyed in any coral. We set out to conduct an evolutionary screen of orthologs in two congeneric acroporid coral species: *Acropora millepora* from the Indo-Pacific and *Acropora palmata* from the Caribbean. We identified a set of 2,604 orthologous cDNA sequences for which we calculated pair-wise *d_N_/d_S_* ratios in order to (i) identify the extent of adaptive evolution in scleractinian corals, and (ii) assess the nature of proteins that are potentially subject to positive selection. Our results indicate that a considerable fraction of coral proteins might be under positive selection, and that TRGs display on average significantly higher evolutionary rates. As such, they might represent important mediators of microevolution and lineage-specific adaptations that warrant further examination for assessing the future response of corals to a changing environment.

## Results

### Evolutionary Screen

Out of 99,091 assembled unique sequences for *A. millepora* and 14,647 unique sequences for *A. palmata*, we gathered 3,295 putative ortholog pairs. We applied several criteria to identify the correct open reading frame (see Materials and Methods, Figure S1), and calculated the ratio of nonsynonymous to synonymous divergence (*d_N_/d_S_* ratio) between both coral species for each putative ortholog pair using PAML [33] as implemented in Pal2Nal [34]. After filtering, we obtained 2,604 putative ortholog pairs. 2,281 ortholog pairs could be annotated according to BLASTx homology searches, while 323 sequences had no significant hit. From 2,604 orthologs, 190 genes showed *d_N_/d_S_* ratios larger than 1. Of those, 68 genes were among the presumably coral-specific (i.e. non-annotated) orthologs (Table 1). This led us to conclude two things: (i) a considerable portion of the orthologs analyzed here show *d_N_/d_S_* values exceeding 1, which is a strong indicator (although not a proof) of positive selection (7% of all orthologs) [23], and (ii) taxonomically-restricted genes had significantly higher *d_N_/d_S_* values (median *d_N_/d_S_* = 0.5040) compared with the annotated set (median *d_N_/d_S_* = 0.2260; *P_MWU_*<0.001). Although the elevated *d_N_/d_S_* ratios of lineage-specific proteins could result from positive selection, they could also result from relaxed selective constraints. Hence, this alone does not constitute evidence for positive selection. The non-annotated set had a significantly higher rate of amino acid substitution (non-annotated median *dN* = 0.0206) compared with the annotated set (annotated median *dN* = 0.0091; *P_MWU_*<0.001), and this elevated rate cannot be attributed to a difference in overall mutation rate as values of synonymous substitutions were similar (non-annotated median *dS* = 0.0416; annotated median *ds* = 0.0426; *P_MWU_* = 0.379). This result confirms that non-annotated proteins evolve faster on average than annotated ones.

**Table 1:** Number of annotated (conserved) and non-annotated (presumably lineage-specific) orthologs.

orthologs	n_orthologs_	%_all_	annotated	%_all_	%_annot_	non-annotated	%_all_	%_non-annot_
all	3295							
filtered	2604	100	2281	88		323	12	
*d_N_/d_S_*<1	2414	93	2159		95	255		79
*d_N_/d_S_*>1	190	7	122		5	68		21

### Evolutionary rate distribution

**Figure 1:**
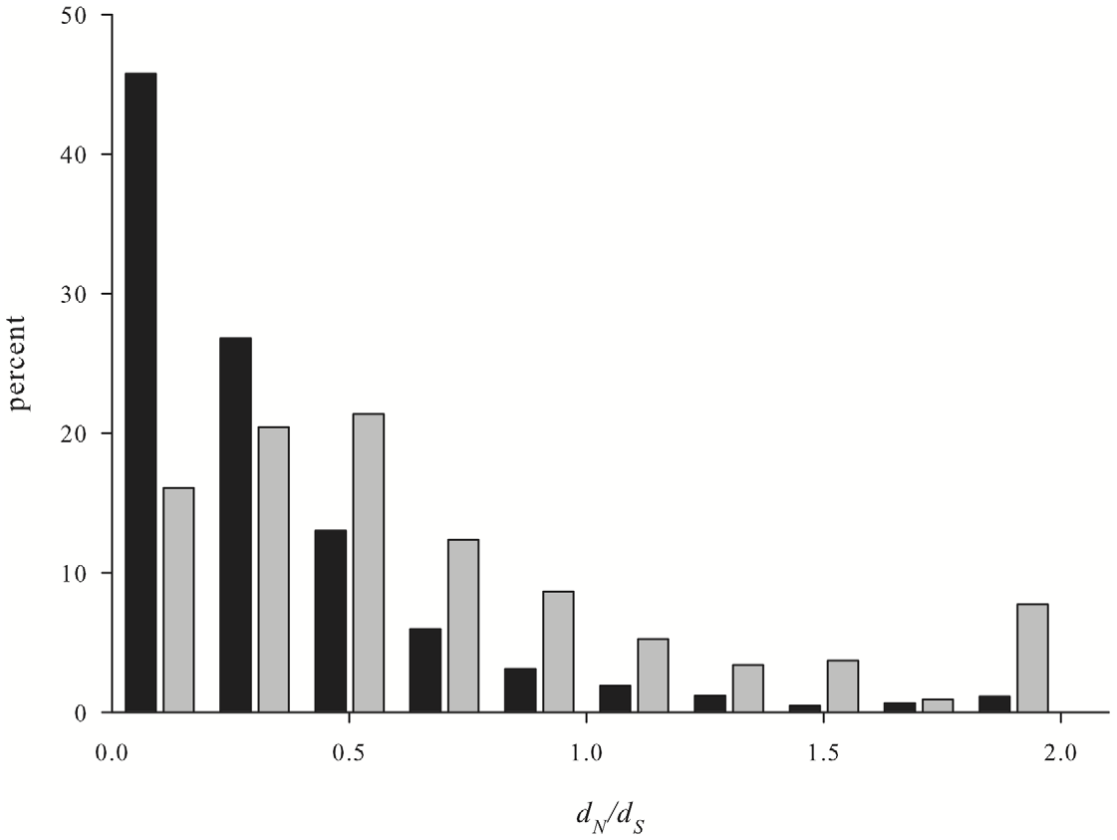
Discrete distribution of *d_N_/d_S_* ratios. The percentages of conserved (black bars) and lineage-specific (grey bars) genes falling into the respective *d_N_/d_S_* classes. Note that *d_n_/d_s_* ratios over 1.8 were pooled for clarity.

Although lineage-specific genes seem to evolve on average significantly faster than annotated genes, there is nonetheless a broad distribution of different rates for both classes (Figure 1). Annotated orthologs were most common at *d_n_/d_s_*<0.5 and successively diminished with increasing *d_n_/d_s_*. In contrast, lineage-specific orthologs were more evenly distributed across *d_n_/d_s_* values between 0 and 0.5, and were in particular present at values >1. We also found potential TRGs with very low divergence rates (*d_n_/d_s_*<0.01), indicative of high conservation. Those genes are particularly interesting as they might have arisen as a result of lineage-specific evolution until they reached an adaptive peak from which further evolution slowed [35]. As a result they are highly conserved between species of the same lineage but cannot be found outside of those lineages. Note that our BLAST-based annotation approach included the cnidarian *Nematostella vectensis*, so the TRGs are actually restricted even within cnidarians.

### Expression of *d_N_/d_S_* orthologs

The nature of TRGs does not allow for assigning functions based on homologies. Hence, it is not possible to compare *ad hoc* the functional distribution of conserved (i.e. annotated) and lineage-specific (i.e. non-annotated) orthologs. However, expression can be used as a first proxy to the function of a gene [36]. To this date, several studies used microarray expression profiling and whole mount in situ hybridization in corals to identify conserved and lineage-specific genes that play a role in development, bleaching, symbiosis, and heat stress [7], [8], [9], [11], [37], [38], [39]. Grasso *et al.* [38] conducted a microarray analysis of coral development in which they analyzed four major stages of coral development in *A. millepora* (prawnchip, planula, polyp, and adult stage). They were able to identify six major synexpression clusters that mapped onto the four stages of coral development. In order to compare expression between our set of conserved and lineage-specific orthologs, we mapped orthologs from *A. millepora* to the sequences that were contained in the expression clusters from the Grasso dataset via BLASTn. In each life stage, we identified the majority of the genes that were originally assigned to a cluster among our set of *A. millepora* orthologs (although our *A. millepora* EST dataset was derived from planulae larvae) (Table 2). First, we found no significant overrepresentation of lineage-specific genes in any of the clusters ((χ^2^-test, *P* = 0.224). Next, we conducted a Two Way ANOVA on log_10_-transformed *d_N_/d_S_* ratios with cluster and annotation as the dependent variables. As expected, there was a significant difference in the mean *d_N_/d_S_* values between the annotated and non-annotated orthologs (*P*<0.001). There was, however, no significant difference between clusters (*P* = 0.295), and also no significant interaction between clusters and conserved or lineage-specific orthologs (*P* = 0.666).

**Table 2:** Expression of *d_N_/d_S_* orthologs across developmental stages of *Acropora millepora*.

Cluster *sensu* Grasso *et al.*	expression	n	n_orthologs_	%	n_conserved_	n_coral-specific_	AV *d_N_/d_S_* conserved orthologs	AV *d_N_/d_S_* lineage-specific orthologs
I	prawnchip	567	417	73.5	372	45	0.32	0.65
II	planula	110	86	78.2	76	10	0.38	0.76
III	planula/polyp	159	121	76.1	115	6	0.36	0.71
IV	polyp	77	65	84.4	61	4	0.44	0.67
V	adult	43	32	74.4	29	3	0.35	0.37
VI	planula/polyp/adult	205	155	75.6	146	9	0.32	0.82
						AV	0.36	0.66

### Functional distribution of orthologs with elevated *d_S_*, *d_N_*, and *d_N_/d_S_*

**Figure 2:**
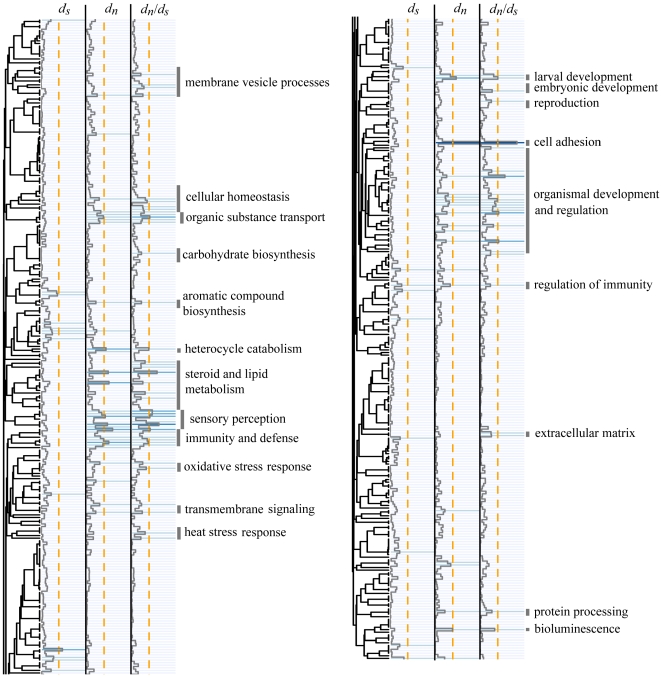
Detection of biological processes experiencing accelerated protein sequence evolution. The dendrogram reflects the proportion of orthologs shared between different categories in our dataset (see Material and Methods). The colors of the corresponding cells and the overlying trace line represent *P*-values of Mann-Whitney *U* test for elevated *d_S_*, *d_N_*, and *d_N_/d_S_* values. The first transition to the darker color signifies *P*<0.05 in an individual comparison. The dashed orange line indicates the 10% false discovery rate cutoff.

**Figure 3:**
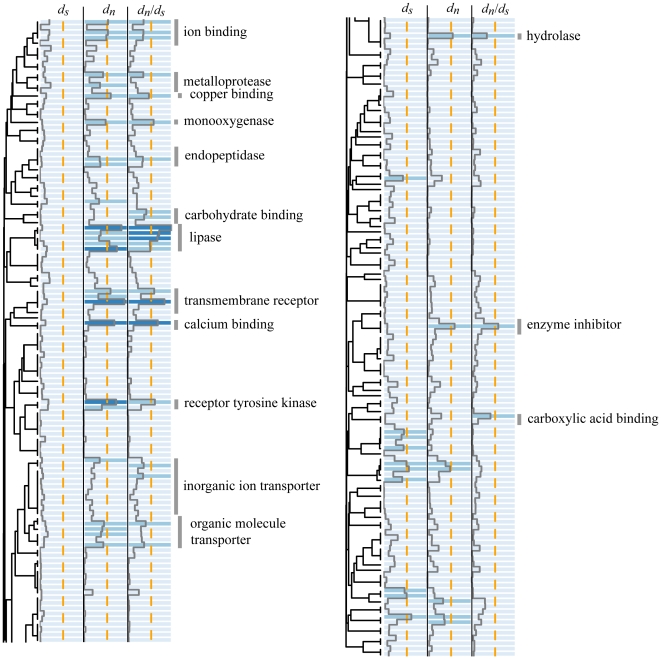
Detection of molecular functions experiencing accelerated protein sequence evolution. The dendrogram reflects the proportion of orthologs shared between different categories in our dataset (see Material and Methods). The colors of the corresponding cells and the overlying trace line represent *P*-values of Mann-Whitney *U* test for elevated *d_S_*, *d_N_*, and *d_N_/d_S_* values. The first transition to the darker color signifies *P*<0.05 in an individual comparison. The dashed orange line indicates the 10% false discovery rate cutoff.

We applied Mann-Whitney *U* (MWU) test to see whether the indices of evolutionary rates were distributed unevenly across functional categories, based on annotations established using Gene Ontology (GO) terms for “biological process” and “molecular function”. Plotting the MWU test *P*-values across GO categories (Figures 2 and 3, Figures S2 and S3) indicated that the observed *d*_N_/*d*_S_ variation is predominantly driven by variation in *d_N_* rates (as expected under varying selection pressures). We visualized a number of functional clusters showing a tendency to rank higher than the rest of the dataset with respect to *d_N_/d_S_*. Several of the highlighted GO categories passed the 10% false discovery rate cutoff [40] (Table 3).

**Table 3:** List of GO categories most strongly enriched for orthologs with elevated *d_N_/d_S_*.

Adjusted *P*_MWU_ *	GO term	Description
*Biological process*:		
0.002	GO:0007155	cell adhesion
0.021	GO:0003008	system process
0.027	GO:0006629	lipid metabolic process
0.027	GO:0032501	multicellular organismal process
0.070	GO:0007600	sensory perception
0.070	GO:0007601	visual perception
0.087	GO:0002009	morphogenesis of an epithelium
0.087	GO:0002376	immune system process
0.087	GO:0050877	neurological system process
0.087	GO:0071702	organic substance transport
0.087	GO:2000026	regulation of multicellular organismal development
0.099	GO:0002164	larval development
0.102	GO:0046700	heterocycle catabolic process
0.102	GO:0051239	regulation of multicellular organismal process
*Molecular function*:		
0.010	GO:0016298	lipase activity
0.026	GO:0004888	transmembrane receptor activity
0.053	GO:0004620	phospholipase activity
0.053	GO:0005509	calcium ion binding
0.069	GO:0004714	transmembrane receptor protein tyrosine kinase activity
0.069	GO:0004857	enzyme inhibitor activity
0.069	GO:0004872	receptor activity
0.070	GO:0004497	monooxygenase activity

*P-value of Mann-Whitney *U* test, adjusted according to the false discovery rate method (cutoff 10%) [40].

### Candidate genes with potential relevance to cnidarian-dinoflagellate symbioses that display elevated rates of evolution

Many biological systems rely on symbiotic interactions between different organisms. Coral reef ecosystems, in particular, depend on the mutualistic relationship between corals and their endosymbiotic, dinoflagellate algae. Here, we generated a candidate gene list through literature perusal (and GO categories therein) containing homologs that are likely to be of relevance to cnidarian-dinoflagellate symbioses and that displayed elevated rates of evolution (Table 4). Among these, we found many members from the cellular stress-, heat stress-, and antioxidant-response system. Genes related to the innate immune system and sugar-binding proteins gave rise to a partial gene inventory (Table 4). Other genes that are likely to play a role in the cellular events surrounding the breakdown of symbiosis (exocytosis, apoptosis and/or autophagy [41], [42], [43], [44], [45], [46], [47]) were also identified.

**Table 4:** Potential symbiosis-related genes displaying elevated rates of evolution identified from the transcriptomes of *A. millepora* and *A. palmate.*

Name	*A. millepora* SymBioSys ID	SwissProt Accession	E-value BLASTx	*d_N_/d_S_*	Notes
**stress-/heat stress-/oxidative stress-/antioxidant activity-related**					
Glutaredoxin-1	SEQINDEX4598_C_c	GLRX	6.00E-15	1.90	cell redox homeostasis
Glutathione S-transferase omega-1	SEQINDEX4951_C_c	GSTO1	2.00E-09	1.73	oxidative stress response
Peroxidasin homolog	SEQINDEX7200_C_c	Pxdn	3.00E-81	1.65	oxidative stress response
Protein LSM14 homolog A	SEQINDEX12122_C_c	LSM14A	2.00E-32	1.07	
Endoplasmic reticulum resident protein 44	SEQINDEX14162_C_c	Txndc4	1.00E-100	1.01	
Heat shock protein Hsp-16.2	SEQINDEX2354_C_c	hsp-16.2	5.00E-13	0.97	stress response
**Apoptosis-/Autophagy-related**					
Apoptosis regulator BAX	SEQINDEX5561_C_c	Bax	2.00E-18	1.09	
**Endo-, Exo-, Phagocytosis-related**					
CD63 antigen	SEQINDEX6578_C_c	CD63	1.00E-29	2.31	growth regulation
MIT domain-containing protein 1	SEQINDEX12663_C_c	MITD1	9.00E-45	1.25	endosomal protein transport
Gamma-glutamyl hydrolase	SEQINDEX13089_C_c	GGH	1.00E-31	1.21	
Synaptosomal-associated protein 29	SEQINDEX2151_C_c	SNAP29	3.00E-21	1.20	cellular membrane fusion
**Immunity-related**					
Gamma-IFN-inducible lysosomal thiol reductase	SEQINDEX2720_C_c	IFI30	1.00E-30	1.42	innate immune response
Ectonucleoside triphosphate diphosphohydrolase 1	SEQINDEX4337_C_c	ENTPD1	2.00E-40	1.37	regulates homotypic adhesion
Lipopolysaccharide-binding protein	SEQINDEX3027_C_c	LBP	5.00E-55	1.03	binds to bacterial lipopolysaccharides (LPS), Toll signaling pathway
Toll-like receptor 2	SEQINDEX6872_C_c	TLR2	4.00E-13	0.98	mediates innate immune response to bacterial lipoproteins

## Discussion

### Evolutionary Screen

A major factor that comes into play when assessing *d_N_/d_S_* ratios is that with higher evolutionary divergence, *d_S_* becomes saturated with multiple substitutions per site on long branches. Hence, neutral evolution is underestimated and, as a consequence, comparisons between different species are only valid within a given divergence range. The genus *Acropora* (Scleractinia: Acroporidae) is one of the most widespread genera of corals as it spans the Indian and Pacific Oceans and the Caribbean Sea. It is also the largest extant reef-building coral genus with numbers of species estimates ranging from 113 to 180 [48]. In this study, *A. millepora* is representing a member of the Indo-Pacific *Acropora* species and *A. palmata* is representing a member from the Caribbean. Molecular analyses suggest that *A. millepora* and *A. palmata* had their latest contact around 12 Myr ago, while Indo-Pacific *Acropora* species have radiated over the last 10 Myr [48]. If we assume a generation time of 1 to 10 years [49], [50] and a mutation rate of 10^−8^ per nucleotide site per generation for both species [51], we come up with the following proxy for *d_S_*: 10^7^ generations (divergence time/generation time) * 10^−8^ (mutation rate) = 0.1. Hence, we expect an average divergence at neutrally evolving sites of approximately 10% (given that both species have the same mutation rate). This estimate is the same order of magnitude as the median *d_S_* of our set of orthologs (median *d_S_* = 0.043), and consequently our approach does not seem to inflate measures of *d_S_*. Even for genes that evolve fast, this divergence time frame allows one to identify the respective ortholog in both species.

We found that a considerable portion of the orthologs showed *d_N_/d_S_* values exceeding 1 (7% of all orthologs), and that TRGs had significantly higher *d_N_/d_S_* values. This finding might indicate that the group of TRGs plays a vital role in adaptive evolution. These genes did not show homology anywhere, including the sea anemone *Nematostella vectensis*, which belongs to the same subclass but a different order (subclass Hexacorallia, order Actiniaria). Although many of these genes may be coral-specific (i.e., restricted to stony corals, order Scleractinia), we cannot rule out that they are present in other, currently unsampled, orders of Hexacorallia (e.g. Corallimorpharia and Zoanthidea), or even have a broader pan-Anthozoan distribution but happen to be missing in the sea anemone. Studies in *Drosophila* have shown that TRGs represent a group of genes that on average display higher *d_N_/d_S_* ratios and are likely to play an important role in lineage-specific adaptations [35], [52]. Furthermore, a recent study on orthologs from coral symbionts, *Symbiodinium* spp., identified the highest *d_N_/d_S_* ratio in a *Symbiodinium*-specific gene, and the authors speculated accordingly that the portion of genes with elevated *d_N_/d_S_* values might be higher in the group of lineage-specific genes in comparison to conserved genes [53]. The authors further hypothesized that a symbiotic lifestyle might affect sequence evolution, as genes might need to coevolve with their symbiotic partners.

The ability to differentiate between self and non-self plays a particular role for reef-building corals in light of their mutualistic, intracellular symbioses with dinoflagellate algae as these need to be distinguished from other eukaryotic protists (dinoflagellates are alveolates and a sister group to the apicomplexans – obligate intracellular parasites – that may use the same receptors and signaling pathways to gain access to the host cell). In addition, competition between different symbiont strains might facilitate the evolution of genes involved in recognizing different clades of *Symbiodinium*, which often can associate with the same coral species [54]. A recent study suggested that transcriptomic states of the Caribbean coral *Montastraea faveolata* (a coral that can host multiple *Symbiodinium* genotypes) were correlated with differences in the *Symbiodinium* genotype hosted [55]. It will be interesting to test whether the percentage of genes under adaptive evolution is higher in corals that are able to host multiple versus only one genotype of *Symbiodinium*. Given that the generation time of *Symbiodinium* spp. is orders of magnitude smaller than those of corals, selection in corals might act on being less discriminating between different algal types that in turn evolve to cope with a changing environment.

### Expession of *d_N_/d_S_* ortholgs

By definition, functional inference by homology to known genes is not available for TRGs. However, expression of those genes might indicate and serve as a proxy for functional significance. Given that some of the TRGs are likely a result of lineage-specific adaptations, it seems plausible to assume that they are expressed specifically with regard to life stage as 1) they presumably have specialized functions, and 2) restricted expression tends to minimize pleiotropic interference. Here, we wanted to test whether specific life-stages show an enrichment or depletion of high *d_N_/d_S_* genes in the group of annotated and non-annotated genes. While we were not able to show statistically significant differences, our data indicates that the adult life stage of corals has a similar *d_N_/d_S_* distribution for conserved (i.e. annotated) and coral-specific (i.e. non-annotated) genes (Table 2). By contrast, all other life stages show a higher mean *d_N_/d_S_* in supposedly coral-specific genes in comparison to conserved genes (Table 2). This might indicate that in order to better understand and investigate adaptive evolution of corals, particular attention has to be paid to non-annotated genes, and that expression of these genes are more easily found in life stages other than adult.

It is interesting to note that we found lineage-specific genes with low *d_N_/d_S_* values that showed stage-specific expression (e.g., lineage-specific orthologs in the adult stage). Those genes either arose *de novo* [56], through gene duplication and subsequent diversification [35], or were retained from a common ancestor but lost elsewhere [57]. They likely represent genes that support coral-specific adaptations, as they are conserved among corals but not found outside this lineage. A promising approach that arises from these considerations is that slowly evolving TRGs are enriched for “coral-specific” processes and are expressed stage-specifically. A combination of *in silico* and *in situ* approaches that couples evolutionary analyses with signatures of expression might prove a useful strategy to target such genes for further functional studies.

### Functional analysis of the variation in protein evolution rates

Analysis of all the functional categories represented within our set of orthologs suggested a number of processes that experience accelerated rates of protein evolution (Figures 2 and 3, Table 3). Although many of these signatures may be due to relaxed purifying selection rather than positive selection, we detected anticipated targets of the latter. For example, proteins involved in immunity and defense, reproduction, and sensory perception (including transmembrane receptors and associated signaling pathways) are under positive selection in a wide variety of animals, from primates [22] and other mammals [58] to fruit flies [52]. “Bioluminescence” category in our case contained GFP-like fluorescent proteins, which have been shown to experience strong positive selection in corals [16]. In addition to these “usual suspects”, we saw elevated rates of evolution in several other functional groups that are not highlighted in studies of other animals and may therefore reflect the specifics of coral evolution. Some of these are ostensibly related to the corals' endosymbiotic relationship with *Symbiodinium* dinoflagellates, such as management of membrane vesicles, transmembrane transport of ions and organic molecules, and cellular homeostasis (which includes the category “maintenance of cellular location”). The category that was the most enriched with rapidly evolving proteins – cell adhesion – may also be related to symbiosis, as its members are linked to a number of cell surface molecules that may mediate host-symbiont recognition (Table S1). These proteins are expected to evolve under positive selection due to the need for frequent specificity readjustments and potentially due to “arms race” between the coral and cheater (i.e. non-compatible) strains of *Symbiodinium*.

Some of the functions highlighted by our analysis were rather unexpected. Most notably, proteins involved in metabolism of lipids and steroids feature prominently in both biological process and especially molecular function analyses (Table 3, Table S1, Table S2), for which we are not yet ready to offer a biological explanation. Some other functional groups may appear as rapidly evolving due to sharing of orthologs with other GO categories (reflected by the dendrogram in Figures 2 and 3). For example, “multicellular organismal development” may have become highlighted in our analysis due to substantial sharing of orthologs with the “cell adhesion” category. More generally, the inherent redundancy of the GO database leads to partial overlaps in the outcomes of different categories, so that any result on functional analyses based on GO annotations (irrespective of the methodology) must be viewed as the union of all possible interpretations of the data. Since only one of these interpretations is correct, some false positives are unavoidable. Selecting the correct interpretation would be possible based on additional systems biology data, which is still lacking for corals but may become available in the near future from whole-transcriptome expression profiling studies.

### Candidate genes with potential relevance to cnidarian-dinoflagellate symbioses that display elevated rates of evolution

Since the global GO analysis may not adequately reflect the mechanisms specific for coral biology, we looked at a set of candidate genes that were either directly implicated in cnidarian-dinoflagellate symbiosis by empirical evidence, or functionally interconnected with them in molecular pathways. Within this gene set, proteins that play a role in corals' response to stress and genes related to immunity were the most prominently represented ones.

Stress-induced photoinhibition and damage to the algal photosystem II are thought to be responsible for an increased production of reactive oxygen species [59], [60], and consequently, diffusion of hydrogen peroxide (H_2_O_2_) through the membranes into the host cell(s) [61]. H_2_O_2_ then activates a cellular cascade, which results in expulsion of symbionts and bleaching [62]. The molecular pathways in the coral host to prevent bleaching (i.e. heat stress and oxidative stress) might therefore be under positive selection in order to mitigate the effects of stress on the coral-algae symbiosis. Consequently, many of the stress genes we identified (Table 4) were identified as differentially expressed in recent microarray studies on heat stress and bleaching in corals [7], [8], [10], [11]. Among the stress-related genes, we detected elevated rates of evolution in Hsp-16.2, Glutaredoxin-1, Glutathione S-transferase omega-1, and a Peroxidasin homolog (Table 4).

Genes related to innate immunity gave rise to another partial inventory of rapidly evolving genes. From the gene expression regulation standpoint, coral-algae specificity seems to arise not from the fact that a coral responds to an appropriate symbiont strain, but from active exclusion of other strains through immunity and apoptosis [37], [63]. Evolution of association with novel algal strains could therefore be enabled by mutations in recognition receptors typically responsible for their exclusion, such as immunity genes. Several genes thus far implicated in the establishment of coral-algae partnerships may indeed be broadly responsible for allorecognition and immune response regulation, such as glycans and lectins [64], [65], fasciclin [66], and MAPK-kinase and NF-kappa-B [67]. The latter two genes regulate antimicrobial response in invertebrates [68], which is somewhat different from their function in mammals. In this study, Gamma-IFN-inducible lysosomal thiol reductase, lipopolysaccharide-binding protein, and Toll-like receptor 2, all implicated in the innate immune response to bacterial pathogens, displayed elevated rates of evolution.

While our evolutionary screen between two coral species allowed for the delineation of fast-evolving functional categories, ultimately one is interested in identifying the specific genes and amino acid sites that are under adaptive evolution. Conducting similar analyses in a multi-species framework will make it possible to investigate this question in a robust statistical framework, allowing for amino acid site- and species-specific identification and characterization of positively selected genes. However, at the moment we have next to no information about the evolutionary mechanisms that brought about morphological, ecological, and physiological diversity of corals. This study provides an initial birds-eye view of genome-wide evolutionary patterns in corals and will serve as a guide for subsequent studies focusing on finer details of adaptation. Some of the genes that we highlighted in this initial screen may be responsible for thermal adaptation and therefore be targets of natural selection driven by increasing seawater temperatures as a consequence of climate change. They therefore represent a meaningful set of genes providing working hypotheses to look for genetic markers of climate change-driven evolution.

## Materials and Methods

### EST libraries, sequencing, assembly, and annotation

The datasets used in this study include an expanded version (see below) of a Sanger EST dataset from *Acropora palmata* [17] and a normalized 454 EST dataset from *Acropora millepora* [69]. *Acropora millepora* sequencing reads have been deposited in NCBI's SRA database, along with the assembly output (accession: SRA003728). *Acropora palmata* sequences are deposited in GenBank with the accession numbers DR982333–DR988505, EY021828–EY031784, FE038910–FE040597, DR982333-DR986349, EY021857-EY031784, and GW189124-GW218328. All sequences are available from the SymBioSys database at http://sequoia.ucmerced.edu/SymBioSys/index.php. For *A. palmata*, 29,205 additional ESTs were generated from a pooled EST library, which included RNA from unfertilized eggs, various larval stages, heat- and light-stressed larvae, and heat-stressed adult fragments. This library was normalized and sequenced at the DOE-Joint Genome Institute. Both datasets were processed as described in [70] using our EST pipeline and database SymBioSys (http://sequoia.ucmerced.edu/SymBioSys/index.php). Briefly, all unique sequences were annotated by a BLASTx homology search (E<10^−5^) against the UniProt, Swissprot, and TrEMBL databases. We denoted a gene as lineage-specific or taxonomically-restricted if the sequence did not yield a BLASTx hit to the TrEMBL database with an e-value smaller than 10^−5^. All raw, assembled, and annotated sequences are accessible as ‘Amil_v2’ and ‘Apal_EST’ through the SymBioSys database.

### Identification of putative orthologs by Best Reciprocal BLAST Hit approach

Putative orthologs in *A. millepora* and *A. palmata* were identified by a Best Reciprocal BLAST Hit approach using the two assembled EST datasets described above (Figure S1). Initially, the method was developed as a shortcut to identify orthologs between genomes [71], [72], but is assumed to work equally well for EST sequences [73]. Briefly, all-against-all BLASTs of both datasets were conducted to obtain a library-specific best hit. We applied this method by identifying Best Reciprocal tBLASTx Hits with a bitscore cutoff of 300 for any given alignment (Table S3). Since tBLASTx hit alignments of translated nucleotide sequences may not necessarily correspond to the correct reading frame of the nucleotide sequences, we applied a series of tests to reduce the number of falsely calculated *d_N_/d_S_* ratios (see below). First, the correct orientation (5′ to 3′) of all *A. palmata* sequences was known, since all cDNAs were directionally cloned [17]. Accordingly, all *A. millepora* sequences were also oriented in the forward direction as determined from a BLASTn against the corresponding *A. palmata* sequence. Second, all *A. millepora* sequences were annotated according to Uniprot TrEMBL protein database entries using BLASTx (E<10^−5^). For those sequences that had BLASTx hits, we parsed the longest and second longest stop codon-free protein sequence (LSCFPS and 2^nd^ LSCFPS) from the tBLASTx alignments and searched for homologs of both sequences in the Uniprot TrEMBL database using BLASTp (E<10^−5^). If either the LSCFPS or the 2^nd^ LSCFPS BLASTp hit was identical to the BLASTx hit of the nucleotide sequence, we were highly confident that we identified the correct reading frame. If neither the LSCFPS nor the 2^nd^ LSCFPS matched the BLASTx hit, we excluded the putative ortholog pair from further analysis. For non-annotated sequences, the alignment of the LSCFPS or the 2^nd^ LSCFPS was called correct, if both nucleotide sequences for the LSCFPS or the 2^nd^ LSCFPS were oriented in the forward direction, respectively. Again, the putative ortholog pair was excluded from further analysis if none of these rules applied to the tBLASTx alignments (Figure S1). Finally, sequences with homology search hits to mitochondrial sequences were removed. This was done by conducting BLASTn searches of all orthologs against the mitochondrial genome of *Acropora tenuis* (GenBank NC_003522).

### Evolutionary Screen and Data analysis

We tested for evidence of positive selection by comparing the nonsynonymous substitution rate (*d_N_*) to the synonymous substitution rate (*d_S_*). Briefly, protein sequences of each ortholog pair were parsed from the tBLASTx results and aligned using ClustalW [74] with default parameters. The aligned protein sequences and corresponding nucleotide sequences were used as input files for Pal2Nal [34], which generates codon alignments and calculates gene-wide *d_N_/d_S_* values based on the codeml program implemented in PAML (Model M0) [75], [76] (Table S3). As *d_N_/d_S_* values for multiple mutations at a single site are not reliable, we chose to exclude those ortholog pairs that had *d_N_* or *d_S_* estimates greater than one. In addition, all *d_N_/d_S_* ratio estimates of 99 were removed from further analyses. To assess statistical significance of the difference in evolutionary rates between conserved and lineage-specific sets, we conducted Mann-Whitney *U* tests on *d_S_*, *d_N_*, and *d_N_/d_S_* distributions.

### Functional analysis of accelerated protein evolution

Gene ontology (GO) [77] annotations were assigned to the ortholog pairs based on their best match to the UniProt database [78] and expanded based on the current GO hierarchy (as of March 18, 2011, http://www.geneontology.org/ontology/obo_format_1_2/gene_ontology.1_2.obo) to include all parents of the initially assigned terms. All subsequent calculations and plotting were performed in R [79]. The categories that were either represented by less than 5 orthologs or contained more than 25% of all orthologs were discarded; redundant categories (containing identical sets of orthologs) were removed to leave a single category with the most specific GO level. For “biological process” division, this filtering resulted in 1,426 orthologs in 502 GO categories, for “molecular function” division, the result was 1,433 orthologs in 239 GO categories. We then performed hierarchical clustering of GO categories following Kosiol *et al.* [58]. Specifically, the similarity of each pair of categories was calculated as the number of the shared orthologs divided by the size of the smaller of the two compared categories. The clustering was performed using a matrix of pairwise dissimilarities (1-similarity) using *hclust* function, method “average”. Such clustering simplifies the extensive hierarchy of the GO database, resulting in easily interpretable GO grouping tailored to the particular sequence dataset.

To avoid imposing arbitrary thresholds when selecting orthologs for GO analysis, we analyzed the distribution of *d_S_*, *d_N_*, and *d_N_/d_S_* values across all categories that passed our filters using one-sided Mann-Whitney *U* test, following the approach proposed earlier [58], [80]. The test identified categories containing orthologs that ranked significantly higher than the rest with respect to *d_S_*, *d_N_*, or *d_N_/d_S_*. The resulting *P*-values were plotted (after -log_10_ transformation) as a heat map (function *heatmap.2*) using the result of hierarchical clustering of GO categories as the grouping dendrogram. The custom color palettes for the heat maps were generated using function *brewer.pal*, such that the first transition to the darker color would correspond to *P*<0.05 in an individual test for a particular GO category. Thresholds at which the result would pass the 10% false discovery rate [40] cutoff were determined using function *p.adjust*. The GO terms in the resulting heat maps were manually summarized to indicate functional modules showing signatures of accelerated evolution. The heat maps listing descriptions for all represented GO categories, as well as full listing of annotated orthologs comprising the GO categories passing the 10% false discovery rate threshold, are available in supplementary data (Figures S2, Figure S3, Table S1, Table S2).

## Supporting Information

Figure S1 **Identification of putative orthologs between** ***A. millepora*** **and** ***A. palmata*** **by Best Reciprocal BLAST Hit and subsequent filtering approach.**
(TIFF)

Figure S2 **Detection of biological processes experiencing accelerated protein sequence evolution.** The dendrogram reflects the proportion of orthologs shared between different categories in our dataset (see methods). The colors of the corresponding cells and the overlying trace line represent *P*-values of Mann-Whitney *U* test for elevated *d_S_*, *d_N_*, and *d_N_/d_S_* values. The first transition to the darker color signifies *P*<0.05 in an individual comparison. The dashed orange line indicates the 10% false discovery rate cutoff. The number preceding the definition of a GO category indicates the number of orthologs assigned to this category in our dataset.
(TIF)

Figure S3 **Detection of molecular functions experiencing accelerated protein sequence evolution.** The dendrogram reflects the proportion of orthologs shared between different categories in our dataset (see methods). The colors of the corresponding cells and the overlying trace line represent *P*-values of Mann-Whitney *U* test for elevated *d_S_*, *d_N_*, and *d_N_/d_S_* values. The first transition to the darker color signifies *P*<0.05 in an individual comparison. The dashed orange line indicates the 10% false discovery rate cutoff. The number preceding the definition of a GO category indicates the number of orthologs assigned to this category in our dataset.
(TIF)

Table S1 **List of biological process GO categories most strongly enriched with orthologs (false discovery rate 10%) displaying elevated** ***d_N_/d_S_*** **and their assigned orthologs.**
(XLS)

Table S2 **List of molecular function GO categories most strongly enriched with orthologs (false discovery rate 10%) displaying elevated** ***d_N_/d_S_*** **and their assigned orthologs.**
(XLS)

Table S3 **Orthologs of** ***A. millepora*** **and** ***A. palmata*** **with associated pairwise** ***d_N_/d_S_*** **estimates.**
(XLSX)
