# Supplementary figures and images for: Rapid Evolution of Coral Proteins Responsible for Interaction with the Environment
Source: PLoS One. 2011 May 25;6(5):e20392. doi: 10.1371/journal.pone.0020392 (PMC3102110; doi:10.1371/journal.pone.0020392)

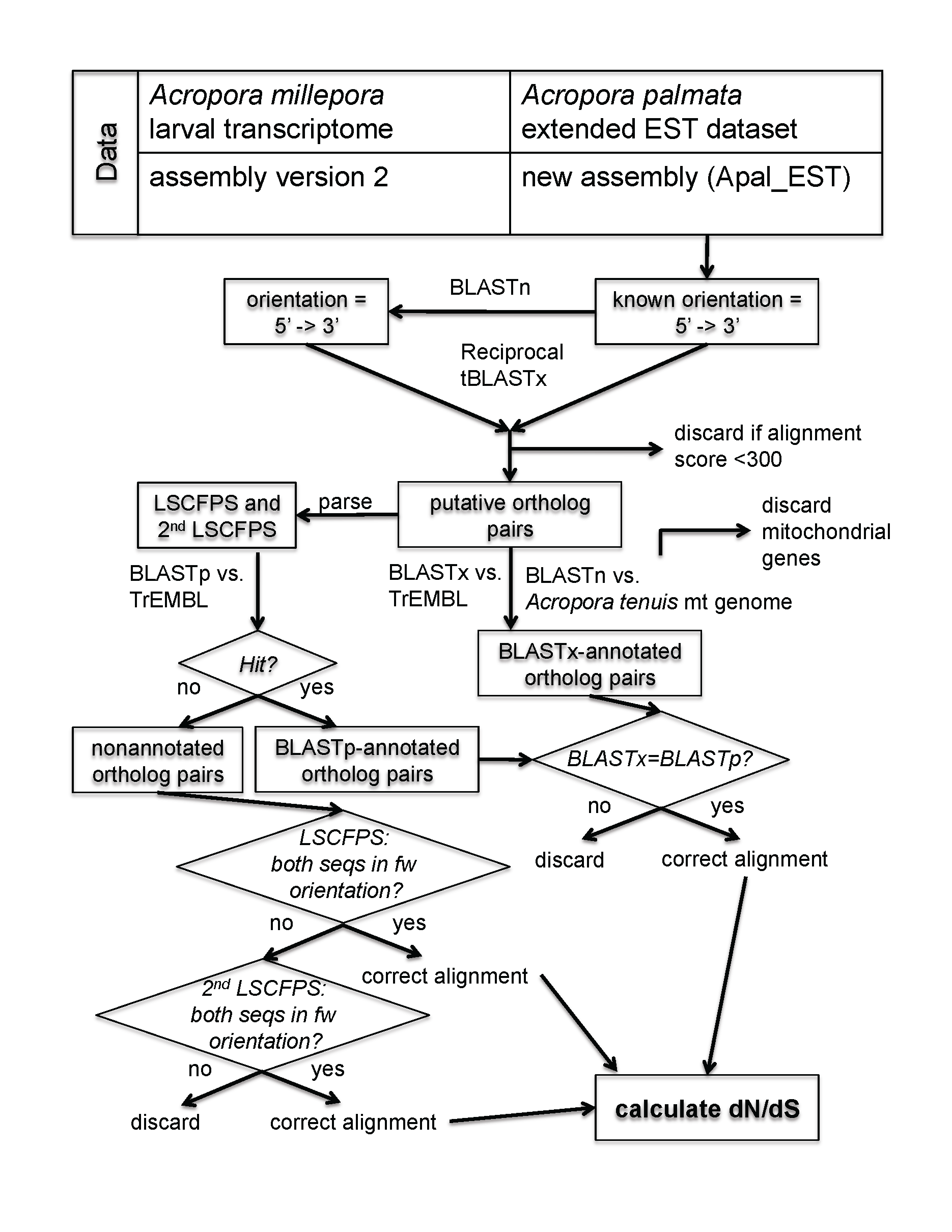

Supplement: Figure S1 — Identification of putative orthologs between A. millepora and A. palmata by Best Reciprocal BLAST Hit and subsequent filtering approach. (TIFF) [file pone.0020392.s001.tiff]

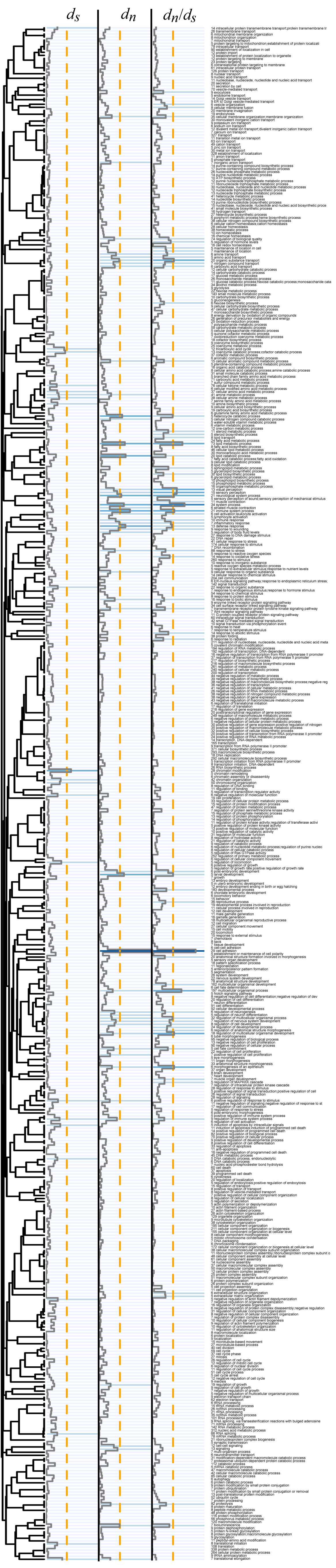

Supplement: Figure S2 — Detection of biological processes experiencing accelerated protein sequence evolution. The dendrogram reflects the proportion of orthologs shared between different categories in our dataset (see methods). The colors of the corresponding cells and the overlying trace line represent P-values of Mann-Whitney U test for elevated dS, dN, and dN/dS values. The first transition to the darker color signifies P<0.05 in an individual comparison. The dashed orange line indicates the 10% false discovery rate cutoff. The number preceding the definition of a GO category indicates the number of orthologs assigned to this category in our dataset. (TIF) [file pone.0020392.s002.tif]

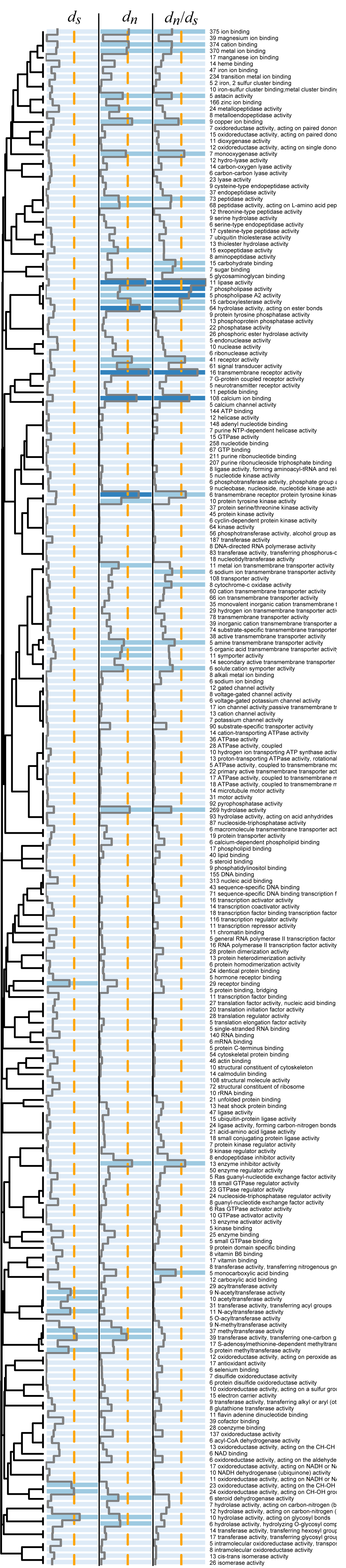

Supplement: Figure S3 — Detection of molecular functions experiencing accelerated protein sequence evolution. The dendrogram reflects the proportion of orthologs shared between different categories in our dataset (see methods). The colors of the corresponding cells and the overlying trace line represent P-values of Mann-Whitney U test for elevated dS, dN, and dN/dS values. The first transition to the darker color signifies P<0.05 in an individual comparison. The dashed orange line indicates the 10% false discovery rate cutoff. The number preceding the definition of a GO category indicates the number of orthologs assigned to this category in our dataset. (TIF) [file pone.0020392.s003.tif]
